# Efficacy of generic teriparatide and alendronate in Chinese postmenopausal women with osteoporosis: a prospective study

**DOI:** 10.1007/s11657-022-01131-8

**Published:** 2022-07-28

**Authors:** Mei Li, Zhenlin Zhang, Qingyun Xue, Qifu Li, Xiaolan Jin, Jin Dong, Qun Cheng, Li You, Hua Lin, Hai Tang, Lin Shen, Xin Gao, Ji Hu, Aijun Chao, Pengqiu Li, Rui Shi, Shuhui Zheng, Ying Zhang, Xiaojiang Xiong, Wei Yu, Weibo Xia

**Affiliations:** 1 Department of Endocrinology, National Health Commission Key Laboratory of Endocrinology, Peking Union Medical College Hospital, Chinese Academy of Medical Sciences, Beijing, China; 2 Department of Osteoporosis and Bone Disease, Shanghai Jiao Tong University Affiliated Sixth People’s Hospital, Shanghai, China; 3 Department of Orthopaedics, Beijing Hospital, National Center of Gerontology, Beijing, China; 4 Department of Endocrinology, The First Affiliated Hospital of Chongqing Medical University, Chongqing, China; 5 Department of Endocrinology, Western Theater Command General Hospital, Chengdu, China; 6 Department of Endocrinology, The First Affiliated Hospital of Shanxi Medical University, Taiyuan, China; 7 Department of Osteoporosis and Bone Disease, Huadong Hospital Affiliated to Fudan University, Shanghai, China; 8 Department of Endocrinology and Metabolism, Shanghai General Hospital, Shanghai Jiao Tong University, 100 Haining Road, Shanghai, China; 9 Department of Orthopaedics, Nanjing Drum Tower Hospital, The Affiliated Hospital of Nanjing University Medical School, Nanjing, China; 10 Department of Orthopedics, Beijing Friendship Hospital, Capital Medical University, No. 95 Yong’an Road, Xicheng District, Beijing, China; 11 Department of Integrated of Traditional Chinese and Western Medicine, Union Hospital, Tongji Medical College of Huazhong University of Science and Technology, Wuhan, China; 12 Department of Endocrinology, Zhongshan Hospital, Fudan University, Shanghai, China; 13 Department of Obstetrics and Gynecology, The Second Affiliated Hospital of Soochow University, Soochow, China; 14 Department of Orthopaedics, Tianjin Hospital, Tianjin, China; 15 Department of Endocrinology, Sichuan Academy of Medical Sciences & Sichuan Provincial People’s Hospital, Chengdu, China; 16 Department of Orthopedics, West China Hospital, Sichuan University, Chengdu, China; 17 Department of Orthopaedics, Heibei General Hospital, Zhengzhou, China; 18 Department of Endocrinology, The Third Affiliated Hospital of Guangzhou Medical University, Guangzhou, China; 19 Department of Orthopedics Joint Disease Area, Chongqing Three Gorges Central Hospital, Chongqing, China; 20 Department of Radiology, Peking Union Medical College Hospital, Chinese Academy of Medical Sciences, Beijing, China; 21 Department of Endocrinology, Key Laboratory of Endocrinology, NHC, State Key Laboratory of Complex Severe and Rare Diseases, Peking Union Medical College Hospital, Chinese Academy of Medical Sciences, Beijing, China

**Keywords:** Teriparatide, Alendronate, Postmenopausal osteoporosis, Bone mineral density, Bone turnover markers

## Abstract

***Summary*:**

The efficacy of generic teriparatide in improving BMD at lumbar spine in patients with osteoporosis was similar to that of alendronate. It provided a new choice for osteoporosis treatment in Chinese population.

**Introduction:**

To determine whether the efficacy of generic teriparatide is noninferior to alendronate for Chinese postmenopausal women with osteoporosis.

**Methods:**

Eligible patients were randomly assigned (2:1) in a 48-week, open-label design to receive 20 µg sc daily teriparatide or 70 mg oral weekly alendronate. Primary outcome was percentage change in BMD at the lumbar spine from baseline to 48 weeks and was assessed for non-inferiority. The same outcome was further assessed for superiority as a secondary endpoint.

**Results:**

Three hundred ninety-one and 196 participants were randomly assigned to the teriparatide or alendronate group, of whom 379 and 194 receiving at least one dose of teriparatide and alendronate treatment were eligible for the efficacy analysis. Teriparatide was non-inferior to alendronate for BMD change at lumbar spine (treatment difference: 0.7%, 95% CI: − 0.3 to 1.7%), which excluded the predefined non-inferiority margin of − 1.5%. However, teriparatide was not statistically superior to alendronate in improving BMD at lumbar spine (*P* = 0.169). At 48 weeks, changes in BMD at total hip were − 1.0% and 2.2% in teriparatide and alendronate group, respectively (*P* < 0.001). The incidence of new fracture showed no statistical difference between groups (*P* = 0.128). Serum P1NP and β-CTX levels significantly increased in the teriparatide group and markedly decreased in alendronate group (all *P* < 0.001 vs baseline). The adverse events (AEs) and serious AEs were more common in the teriparatide group than in the alendronate group, which were mainly teriparatide-related hypercalcemia, elevated alkaline phosphatase or parathyroid hormone, dizziness, and arthralgia.

**Conclusions:**

Teriparatide was not inferior to alendronate in increasing BMD at lumbar spine in Chinese postmenopausal women, and they achieved these effects through different mechanisms.

**Supplementary Information:**

The online version contains supplementary material available at 10.1007/s11657-022-01131-8.

## Introduction

Since the global population is aging, osteoporosis and fragility fracture have become public health concerns because of its considerable morbidity, excess mortality, and great risk of disability. As a country with the largest aging population in the world, China is facing great challenges from osteoporosis and bone fracture [1–3], followed by a great medication burden on the costs of anti-osteoporotic treatment. Although several anti-osteoporosis agents have been approved for treatment of osteoporosis in China, their high costs as original products place a heavy burden not only on individual patients, but also on the healthcare systems [4].

Recently, the domestic generic recombinant human parathyroid hormone (1–34) (teriparatide) has been developed and its safety and preliminary efficacy are confirmed in the phase I and phase II clinical trials. Given its lower price, the domestic generic teriparatide will represent a more affordable treatment option for patients and healthcare systems, thus increasing patients’ access to treatment.

Bisphosphonate therapy is the current standard therapeutic drugs for the prevention and treatment of osteoporosis. Several studies have demonstrated that alendronate is effective in inhibiting bone resorption, increasing bone mineral density (BMD) and reducing fracture risk in postmenopausal women with osteoporosis [5]. Once-daily teriparatide can stimulate bone formation, increases bone mass, and reduces the risk of vertebral and nonvertebral fractures [6]. However, no randomized, controlled trials involving Chinese postmenopausal women with osteoporosis have compared the effects of teriparatide with bisphosphonate.

Here, we conducted this prospective phase III non-inferiority study of the efficacy and safety between domestic generic teriparatide and alendronate in postmenopausal women with osteoporosis in China.

## Methods

### Study design and participants

This was an open-label, phase III, randomized controlled trial conducted at hospitals with clinical pharmacology research qualification in China from June 2014 to April 2016. This study covered 19 large hospitals from different regions of East, South, West, and North China. Baseline characteristics of participants recruited in each center were shown in supplementary Table 1.

Ambulatory postmenopausal women aged 45–85 years old were eligible for enrollment if they had a BMD T-score of − 2.5 or less at the lumbar spine, total hip, or femoral neck or a BMD T-score of − 1.0 or less at either of the above sites with a history of one or more fragility fractures at the vertebra, hip, proximal humerus, or distal radius [7]. Participants were excluded if they had evidence of hyperparathyroidism, hereditary bone diseases or secondary osteoporosis, history of malignancy or radiation therapy, significant cardiopulmonary, hepatic or renal diseases, poorly controlled diabetes or hypertension, gastrointestinal disorders contraindicating alendronate, mental diseases, or cognitive impairment. Participants with treatment history of bisphosphonates within recent 1.5 years before enrolment, or estrogens, selective estrogen receptor modulators within 6 months before enrolment, or calcitonin, glucocorticoids, or vitamin K2 within 3 months before enrolment were also excluded from this study. Additional exclusion criteria included allergy to study drugs or their metabolites, inability to stand or sit upright for at least 30 min.

The study was approved by ethics committee of clinical pharmacology center of Peking Union Medical College Hospital (PUMCH) and all other participating units. All participants provided written informed consents before any study-specific procedure.

### Randomization and treatment

Participants were randomly assigned (2:1) to receive subcutaneous injection of 20ug teriparatide (Suzhou Jinmeng Biotechnology Co., Ltd.) daily or oral 70 mg alendronate sodium (Hangzhou MSD Pharmaceutical Co., Ltd.) weekly. The randomization of participants was not stratified. An interactive web response system (IWRS) was implemented for random allocation at 19 study centers. All participants were supplemented with calcium 500 mg plus Vitamin D_3_ 200 IU (General Electric Pharmaceutical Co., Ltd.) daily. The treatment course was 48 weeks. Participants and investigators were aware of treatment assignment, but BMD assessors, imaging radiologists, and laboratory staff performing biochemical assay were unaware of patients' treatment groups.

### Procedures

Areal BMD of the posterior-anterior lumbar spine, total hip, and femoral neck was measured at baseline, 24 weeks, and 48 weeks of treatments by dual-energy X-ray absorptiometry (DXA) using a Hologic (Hologic, Bedford, MA) or Lunar (GE Healthcare, Madison, WI) equipment. All scans of an individual subject were performed on the same densitometer. Quality control measurements were performed daily with a Hologic or Lunar anthropomorphic spine phantom. DXA scan data were submitted to a central imaging vendor (PUMCH) for blinded analyses. The least significant change (LSC) in BMD measured at lumbar spine was used to evaluate effects on BMD. LSC for lumbar spine obtained at PUMCH was 1.93%.

New incident vertebral fractures were assessed by Genant semi-quantitative grading [8] of lateral X-ray images of spine collected before and after 48-week treatment. The other new fractures were confirmed by medical history and X-ray images during the treatment.

Fasting morning blood samples were obtained at each visit and stored at − 80 °C for batch analyses performed in the central laboratory (PUMCH). Serum procollagen type 1 N-terminal propeptide (P1NP), a marker of bone formation, was measured via electro-chemiluminescent immunoassay (Roche Diagnostics) with inter-and intra-assay coefficients of variation (CVs) of 10% and 8%, respectively. Serum β-C-terminal telopeptide of type 1 collagen (β-CTX), a marker of bone resorption, was measured via a fully automated electro-chemiluminescent immunoassay (Roche Diagnostics) with an inter-assay CV of < 5%. The limit of detection for serum β-CTX was 0.01 ng/mL and the reportable range was 0.01 to 5.99 ng/ml. For each marker, all blood samples from a participant were analyzed together in the same assay run. Bone turnover markers (BTMs) of patients were measured at baseline, 12, 24, and 48 weeks of treatment. Serum levels of calcium, phosphorus, alkaline phosphatase (ALP), alanine aminotransferase (ALT), and creatinine (Cr) were measured by automatic biochemical analyzer.

Data on adverse events (AEs), serious AEs (SAEs), and concomitant medications were collected at each study visit. AEs and SAEs were coded using the Medical Dictionary for Regulatory Activities (MedDRA). At the time of reporting, a physician determined whether AEs were related to the study drug.

### Study outcomes

The primary endpoint of the study was confirmation of non-inferiority of teriparatide to alendronate for the mean percentage change in BMD at the lumbar spine from baseline to 48 weeks. Secondary efficacy end point was superiority test comparing teriparatide and alendronate for the mean percentage change in BMD at the lumbar spine from baseline to 48 weeks, which was evaluated only if noninferiority was demonstrated. Additional end points included the mean percentage changes in BMD at the lumbar spine from baseline to 24 weeks; the mean percentage changes in BMD at total hip from baseline to 24 and 48 weeks; changes in BTMs of P1NP and β-CTX from baseline to 12, 24, and 48 weeks; and the incidence of fracture within 48 weeks of treatment.

### Statistical analysis

The primary hypothesis of this study was that the efficacy of teriparatide was not inferior to alendronate for the mean percentage change in lumbar spine BMD at week 48 from baseline. The secondary hypothesis was that superiority of teriparatide for the mean percentage change in lumbar spine BMD at week 48 from baseline. Based on the previous studies comparing alendronate with placebo [9–12], and the experience of the clinicians who developed the study protocol, it was assumed that the mean change of alendronate treatment at week 48 on the lumbar spine relative to placebo in postmenopausal women was 4.0 ± 3.0%, a sample size of 324 and 162 in the teriparatide and alendronate groups would provide 80% power to detect non-inferiority of teriparatide to alendronate, with a one-sided significance level of 0.025 and non-inferiority margin of − 1.5%. Considering the actual possible shedding of 15%, 382 and 191 patients in the teriparatide and alendronate groups were included in this study. Based on the calculated sample size, the statistical power for the secondary endpoint was 90% to detect a significant superiority of teriparatide for the mean percentage change in lumbar spine BMD at week 48 from baseline.

The efficacy analysis was conducted on the basis of the full analysis set (FAS) and the per-protocol set (PPS). The FAS included all patients who received at least one dose of medication, and the PPS included the patients in the FAS who had no major protocol deviations and received at least 80% of the planned teriparatide or alendronate doses during 48 weeks of treatment. All baseline demographic data was analyzed on the basis of FAS. The data of each visit were described as mean ± SD if normally distributed. Changes in BMD between baseline and each visit were analyzed with repeated measures analysis of variance. The percentage change from baseline in BMD was compared between groups using a linear mixed-effects model with fixed effects for baseline BMD, centers, machine type, and interaction between center and group as covariates. Sensitivity analyses for missing data for percentage change from baseline in BMD at lumbar spine were performed by a repeated measure mixed-effects model. Descriptive statistics of BTMs were expressed as median (interquartile range). The treatment difference of BTMs at each visit was analyzed using Wilcoxon rank sum test. The counting data were presented as frequency (composition ratio). The intergroup comparisons of the incidences of fractures from baseline to 48 weeks were analyzed by Fisher’s exact test.

The safety was evaluated on the safety analysis set (SAS), which included patients who received at least one dose of teriparatide or alendronate. Fisher’s exact test was used to compare the incidence of AEs between groups.

The statistical analyses were carried out using SAS version 9.4 (SAS Institute, Cary, NC, USA) statistical software. *P* values < 0.05 were considered statistically significant.

## Results

**Fig. 1:**
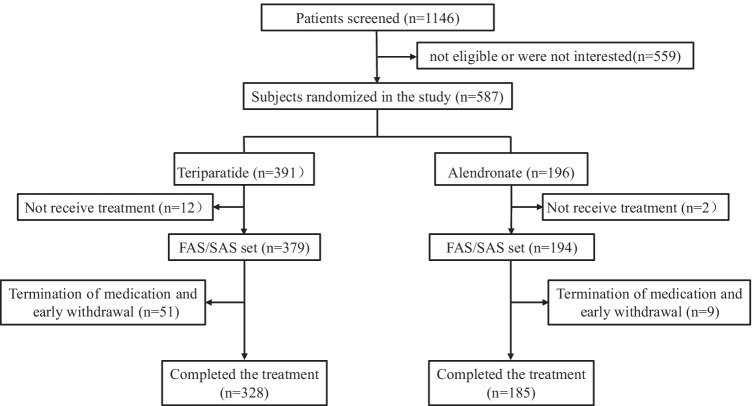
Patient disposition. FAS, full analysis set; SAS, safety analysis set

Of 1146 women who were screened for the study, 559 women were not eligible or were unwilling to participate. Five hundred eighty-seven subjects enrolled in the study were randomly assigned to receive teriparatide (391 subjects) or alendronate (196 subjects) treatment. Five hundred thirteen (87.4%) completed 48 weeks of follow-up, of whom 328 were in the teriparatide group and 185 in the alendronate group. Three hundred seventy-nine in the teriparatide group and 194 in the alendronate group received at least one dose of treatment and were included in the FAS and SAS (Fig. 1). Except for BMD at lumbar spine, there were no differences in baseline characteristics between those who withdrew from the study and completed the study (Supplementary Table 2).

No significant differences were found between these two groups in baseline demographics and clinical characteristics, such as age, height, weight, menopause time, history of osteoporotic fracture, BMD, and BTMs (Table 1).

**Table 1:** Characteristics of teriparatide and alendronate group at baseline

	Teriparatide	Alendronate	*P* Value
	(*n* = 379)	(*n* = 194)	
Age, year, mean ± SD	64.20 ± 7.24	63.68 ± 6.96	0.415
Nationality, *n* (%)			
Han	374 (98.68)	190 (97.94)	0.748
Other	5 (1.32)	4 (2.06)	
Height (cm), mean ± SD	155.10 ± 6.24	154.86 ± 5.81	0.660
Body weight (kg), mean ± SD	55.58 ± 7.29	55.14 ± 7.35	0.488
Time since menopause (year), mean ± SD	15.67 ± 7.90	14.83 ± 7.54	0.234
Comorbidities, *n* (%)	215 (56.88)	97 (50.00)	0.132
Hypertension	102 (26.91)	48 (24.74)	0.576
Dyslipidemia	54 (14.25)	22 (11.34)	0.331
Diabetes	30 (7.92)	11 (5.67)	0.324
Osteoarthritis	10 (2.64)	3 (1.55)	0.558
Others	158 (41.69)	70 (36.08)	0.194
Concomitant medications, *n* (%)	171 (45.24)	87(44.85)	1.000
Chinese patent medicine	132 (34.8)	60 (30.9)	0.197
Antipyretic analgesics	42 (11.08)	25 (12.89)	0.583
Anxiolytics	14 (3.69)	4 (2.06)	0.448
Antihypertensive drugs	14 (3.69)	6 (3.09)	0.711
Previous history of fracture, *n* (%)	130 (34.30)	74 (38.14)	0.363
Vertebral fracture	65 (17.15)	37 (19.07)	0.569
Colles fracture	31 (8.18)	18 (9.28)	0.656
Hip fracture	11 (2.90)	6 (3.09)	0.899
Other fracture	58 (15.30)	25 (12.89)	0.437
BMD, mean ± SD			
Lumbar (L1-4) (g/cm^2^)	0.736 ± 0.103	0.741 ± 0.098	0.550
Total hip (g/cm^2^)	0.709 ± 0.094	0.711 ± 0.096	0.818
P1NP (ng/ml)	56.36(38.16–74.87)	57.69(37.56–79.59)	0.225
β-CTX (ng/ml)	0.36(0.24–0.49)	0.35(0.22–0.51)	0.837
Ca (mmol/L)	2.35 ± 0.10	2.35 ± 0.11	0.986
P (mmol/L)	1.16 ± 0.14	1.16 ± 0.20	0.870
ALP (U/L)	79.37 ± 22.35	82.67 ± 22.25	0.094
Cr (μmol/L)	59.91 ± 10.08	58.61 ± 10.46	0.170
ALT (U/L)	19.68 ± 9.96	20.72 ± 10.06	0.247

*PTH* parathyroid hormone, *n* number, *SD* standard deviation, *P1NP* procollagen type 1 N-terminal propeptide, *β-CTX* β-C-terminal telopeptide of type 1 collagen, *S-Ca* serum calcium, *S-P* serum phosphorus, *ALP* alkaline phosphatase, *Cr* creatinine, *ALT* alanine aminotransferase

### Changes in BMD and incidence of fracture after the treatment

**Fig. 2:**
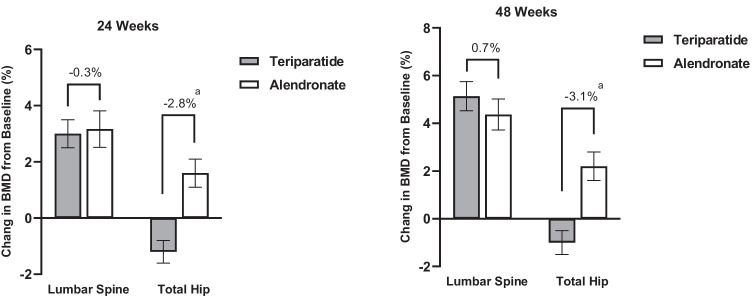
Mean percentage change from baseline in BMD at 24 and 48 weeks. Values represented as mean ± SD,.^a^*P* < 0.001 for between-group comparison

After 24 weeks of treatment, the BMD at lumbar spine and total hip increased 3.0% (95% CI: 2.5–3.5%) and − 1.2% (95% CI: − 1.6 to − 0.8%) in the teriparatide group and 3.2% (95% CI: 2.8–3.8%) and 1.6% (95% CI: 1.1–2.1%) in the alendronate group (all *P* < 0.001 vs baseline) (Fig. 2). Between-group comparisons showed no difference in BMD changes at lumbar spine (*P* = 0.438) while significant difference at total hip (*P* < 0.001). After 48 weeks of treatment, BMD at lumbar spine increased 5.2% (95% CI: 4.6–5.8%) from baseline in the teriparatide group and 4.4% (95% CI: 3.7–5.0%) in the alendronate group. The treatment difference was 0.7% (95% CI: − 0.3 to 1.7%, *P* = 0.169), which excluded the predefined non-inferiority margin of − 1.5%, indicating that the effect of teriparatide treatment was not inferior to alendronate. The superiority test showed that BMD change at lumbar spine between groups did not reach statistical significance (*P* = 0.169). Nevertheless, the PPS analysis showed a greater BMD increase at lumbar spine in the teriparatide group than in the alendronate group (*P* = 0.033). Furthermore, sensitivity analyses for missing data for lumbar spine BMD also showed a significantly greater change in the teriparatide group than in the alendronate group at 48 weeks (*P* = 0.020). BMD at total hip increased − 1.0% (95% CI: − 1.5 to − 0.5%) in the teriparatide group (*P* = 0.001) and 2.2% (1.6–2.8%) in the alendronate group (both *P* < 0.001 vs baseline), with a treatment difference of − 3.1% (95% CI: − 3.9 to − 2.3%) (*P* < 0.001). At 48 weeks of treatment, BMD gains greater than LSC at lumbar spine were 63.43% in the teriparatide group and 63.93% in the alendronate group, with no significant difference between the two groups (*P* = 0.925).

The incidence of new fractures during 48 weeks of treatment was 1.1% (4/377) in the teriparatide group and 2.6% (5/194) in the alendronate group. No statistically significant difference was observed between these two groups (*P* = 0.128).

### Changes in BTMs after the treatment

**Fig. 3:**
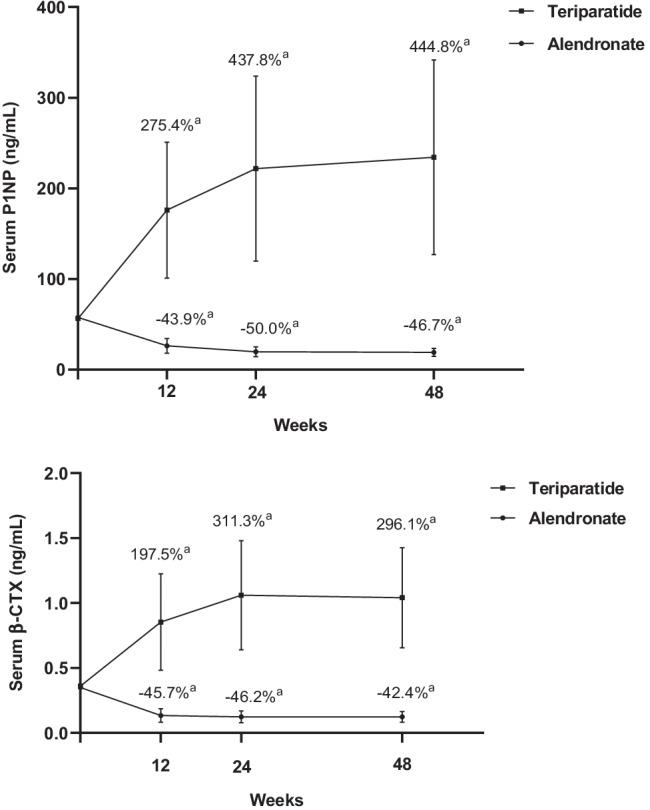
Changes of bone turnover biomarkers after teriparatide or alendronate treatment. Values represented as median (interquartile range),.^a^*P* < 0.001 compared with baseline

Serum levels of P1NP and β-CTX were significantly elevated after 12-, 24- and 48-week treatment of teriparatide (*P* < 0.001 vs baseline). However, serum levels of P1NP and β-CTX significantly declined after 12-, 24- and 48-week treatment of alendronate (*P* < 0.001 vs baseline), which indicated that teriparatide increased BMD through stimulating bone formation while alendronate increased BMD through reducing bone resorption (Fig. 3).

### Adverse events

The overall incidence of any AEs was significantly higher in the teriparatide group than the alendronate group (76.52%% vs. 63.92%%, *P* = 0.002). Drug-related AEs adjudicated by the investigators was 47.76% (181/379) and 26.29% (51/194) in the teriparatide and alendronate groups, respectively, and statistically significant difference was observed between these two groups (*P* < 0.001). Serious AEs were reported in 36 subjects (9.50%) in teriparatide group and 9 subjects (4.64%) in alendronate group (*P* = 0.048). AEs leading to discontinuation of the study occurred in 9 subjects (2.37%) of the teriparatide group, which was more than the alendronate group (1 subject, 0.52%), but not statistically significant (*P* = 0.176). One subject in the teriparatide group died during the study period due to serious trauma caused by traffic accident; the investigator ruled out causal relationship to the study drug.

AEs occurring at an incidence of 5% or above were as follows: elevated ALP (15.04%), hypercalcemia (10.03%), dizziness (8.18%), respiratory tract infection (7.92%), hypercholesterolemia (7.65%), hypertriglyceridemia (6.07%), arthralgia (5.54%), and back pain (5.28%) in the teriparatide group and hypercholesterolemia (8.76%), elevated parathyroid hormone (8.25%), hypertriglyceridemia (7.22%), and upper respiratory tract infection (6.19%) in the alendronate group (Table 2). The incidences of elevated alkaline phosphatase (15.04% vs. 1.03%) and hypercalcemia (10.03% vs. 1.03%) were significantly higher in the teriparatide group than the alendronate group (both *P* < 0.001), which was considered as closely related to the pharmacological action of teriparatide. The adherence in the teriparatide and alendronate groups were 88.73 ± 24.28% and 96.54 ± 14.93%, respectively, with a significant difference between these two groups (*P* < 0.001), which may be due to the fact that once-weekly oral administration is more acceptable than once-daily subcutaneous injection.

**Table 2:** Adverse events during 48 weeks of treatment

Adverse events	Teriparatide (*n* = 379) *n*, %	Alendronate (*n* = 194) *n*, %	*P* value
Any AE	290 (76.52)	124 (63.92)	0.002
Drug-related AEs	181 (47.76)	51 (26.29)	< 0.001
Serious AEs	36 (9.50)	9 (4.64)	0.048
AEs leading to discontinuation of study	9 (2.37)	1 (0.52)	0.176
AEs of interest			
Elevated ALP	57 (15.04)	2 (1.03)	< 0.001
Hypercalcemia	38 (10.03)	2 (1.03)	< 0.001
Dizziness	31 (8.18)	6 (3.09)	0.019
Upper respiratory tract infection	30 (7.92)	12 (6.19)	0.452
Hypercholesterolemia	29 (7.65)	17 (8.76)	0.643
Hypertriglyceridemia	23 (6.07)	14 (7.22)	0.597
Arthralgia	21 (5.54)	6 (3.09)	0.019
Back pain	20 (5.28)	7 (3.61)	0.372
Headache	13 (3.43)	4 (2.06)	0.361
Nausea	10 (2.64)	2 (1.03)	0.335
Elevated PTH	6 (1.58)	16 (8.25)	< 0.001
Death	1 (0.26)	0 (0.00)	1.000

*PTH* parathyroid hormone, *ALP* alkaline phosphatase

## Discussion

In this prospective controlled study, we demonstrated that subcutaneous injection of teriparatide was noninferior to oral alendronate in improving BMD at lumbar spine in postmenopausal women with osteoporosis; moreover, teriparatide showed a trend toward superiority for increasing lumbar spine BMD as well as greater reductions in fracture risks, compared with alendronate during 48 weeks treatment. Teriparatide significantly increased BTMs while alendronate decreased BTMs, indicating the two treatments promote BMD through different mechanisms.

As this study showed, BMD at lumbar spine increased 5.2% after 48-week treatment of teriparatide, which was not inferior to the efficacy of alendronate, but did not meet statistical superiority in FAS analysis. Several randomized trials comparing the efficacy of teriparatide and bisphosphonates found considerably greater BMD increases at lumbar spine in the teriparatide group than in the bisphosphonates group [6, 13, 14]. However, we did not draw this conclusion from this study. Also, a retrospective study suggested that 12 months of teriparatide treatment could lead to a significant 9.7% increase of lumbar spine BMD [15]. A multicenter study assessed the safety and efficacy of teriparatide 20 µg in Japanese men and women with osteoporosis at high risk of fracture and found teriparatide significantly increased lumbar spine BMD by 10.04% at 12 months of treatment [16]. Another study indicated that lumbar spine BMD had increased by 8.1% at 12 months of teriparatide treatment [17]. In the current study, the increase of BMD at lumbar spine with teriparatide treatment was lower relative to the above studies; this was probably because domestic teriparatide was a biosimilar to Forteo®, and the treatment period was relative short. However, if we take PPS into account, there did indeed a statistical greater BMD increase at lumbar spine in the teriparatide group than in the alendronate group (*P* = 0.033); thereby, longer follow-up or larger sample size was needed to further evaluate its clinical efficacy. Meanwhile, we did not observe the total hip BMD increase in the teriparatide group, and the difference with several previous studies also seemed to be related to the shorter treatment time [18–20]. Nevertheless, a study by Susan et al. [21] had found a decrease of 0.5% in total hip BMD at 6 months of teriparatide treatment; also, another study had indicated a slight reduction in total hip BMD of 12 months of teriparatide treatment [22], which were generally in line with the findings in our study. This may be explained by the fact that teriparatide increases bone turnover, then expands the cortical bone remodeling space and replaces older mineralized bone with newer, but not yet fully mineralized bone tissue, thus resulting in a transient decrease in BMD values of cortical bone [23]. The gains of BMD at the proximal femur often require 18–24 months of teriparatide administration [19]; therefore, changes in femoral neck BMD assessed by DXA may not accurately reflect the changes in bone structure or strength that occur with teriparatide in our study.

Osteoporosis occurred as a result of an imbalance between bone resorption and bone formation, with bone breakdown exceeding bone building. Teriparatide had dual, time-dependent effects on bone resorption and bone formation, and intermittent teriparatide could directly increase osteoblasts activity and indirectly increase bone resorption [24, 25]. In this study, teriparatide significantly increased P1NP level, which exceeded the increase of β-CTX, suggesting that bone formation was first stimulated by PTH treatment, and followed by bone resorption. Furthermore, BTMs of P1NP and β-CTX remained in markedly elevated levels, though lower than peak values, at 48 weeks of teriparatide treatment, indicating that bone formation was ongoing increased with teriparatide treatment. In contrast, alendronate has been shown to increase BMD by inhibiting bone resorption. A study assessed the progressive effects of teriparatide and bisphosphonate (zoledronic acid) on bone remodeling and material properties in postmenopausal women with osteoporosis and suggested that teriparatide stimulated new bone formation and produced a mineralized bone matrix with a stable mean mineral content [26], and zoledronic acid slowed bone turnover and prolonged secondary mineralization and produced a highly mineralized bone matrix [26], which revealed that the underlying mechanisms of teriparatide and bisphosphonates increasing BMD were fundamentally different.

Fracture was the most severe complication of osteoporosis. In this study, the incidence of new fracture was 1.1% in the teriparatide group, which was only one half of that in the alendronate group (2.6%), but without significant difference between the two groups. Due to the relative shorter observational period, it was difficult to observe the true effects of teriparatide and alendronate treatment on fracture in this study. Large sample clinical study confirmed teriparatide significantly reduced the risk of new vertebral fracture for patients with all endpoint BMD values, and increases in lumbar spine BMD accounted for 30–41% of the vertebral fracture risk reduction with teriparatide treatment [27]. Data from 4 real-world observational studies showed that teriparatide treatment led to significant reductions in rates of clinical vertebral fracture, non-vertebral fracture, and clinical fractures in patients [28]. A systematic review and meta-analysis of the efficacy of teriparatide in hip fractures in women and men with osteoporosis was conducted, which showed an odds ratio for hip fractures of 0.44 (*P* < 0.05) in patients treated with teriparatide than controls [29]. Therefore, we speculate that teriparatide treatment for a longer period will not only be useful to increase lumbar and hip BMD, but also be of benefit to reducing the risk of vertebral and non-vertebral fractures.

The AEs and serious AEs were more common in the teriparatide group than in the alendronate group. As expected, hypercalcemia and elevated ALP were closely correlated to teriparatide treatment, which would be more frequent in the teriparatide group than in the alendronate group. Also, the frequency of dizziness was significantly higher in the teriparatide group than in the alendronate group, which was consistent with previous studies [30, 31]. This may be related to orthostatic hypotension caused by teriparatide treatment[32]. Except for one patient who died in a traffic accident in the teriparatide group, all serious AEs were gradually relieved during teriparatide treatment. No participants dropped out of the study because of the AEs. However, considering the relatively more common AEs and serious AEs in teriparatide group, it was necessary to closely monitor the teriparatide-related adverse events in its clinical application.

There were several clinical significances of this study. This was the first prospective randomized study to compare the efficacy of domestic generic teriparatide and alendronate in Chinese postmenopausal women with osteoporosis. Studies revealed that teriparatide could significantly increase BMD of lumbar spine, which was not inferior to alendronate. The changes of BTMs fully elucidated the anabolic effects of teriparatide, which was completely different from the mechanism of inhibiting bone resorption of alendronate. The overall safety of domestic teriparatide treatment was well. However, limitations of the study included an open-label design, the relatively short-term observational period, lack of data on BMD changes at femoral neck, and two distinct types of DXA equipment which may lead to bias of results of BMD. The limitations of this study included that it was a relatively short-term follow-up study and the effects of treatment on incidence of fracture were difficult to observe. Also, the lack of blinding in study medication may lead to the relatively higher drop-out rate in the teriparatide group (Fig. 1) due to the inconvenience of daily subcutaneous injection of teriparatide. This high dropout rate might affect study outcomes. Additionally, since BMD was measured using two distinct types of DXA equipment in each study center, the skills of operators and the scanning techniques might have differed across the institutions, which may lead to bias of results of BMD.

In summary, in postmenopausal women with osteoporosis, domestic generic teriparatide could improve BMD at lumbar spine, and the clinical efficacy was non-inferior to alendronate. It provided a new choice for the treatment of postmenopausal osteoporosis in Chinese population, but the clinical research with longer time and larger sample was worth continuing.

## Supplementary Information

Below is the link to the electronic supplementary material.

Supplementary file1 (DOCX 25 KB)

## Data Availability

All data generated or analyzed during this study are included in this published article.
