# Supplementary material for: Efficacy of generic teriparatide and alendronate in Chinese postmenopausal women with osteoporosis: a prospective study
Source: Arch Osteoporos. 2022 Jul 28;17(1):103. doi: 10.1007/s11657-022-01131-8 (PMC9334369; doi:10.1007/s11657-022-01131-8)
Supplement: Supplementary file 1 — Supplementary file1 (DOCX 25 KB) [file 11657_2022_1131_MOESM1_ESM.docx]

|  | Study centers | FAS/SAS, n | Age (year), mean ± SD | Height (cm), mean ± SD | Weight (kg),  mean ± SD |
| --- | --- | --- | --- | --- | --- |
| 1 | Peking Union Medical College Hospital | 23 | 66.89±7.14 | 155.21±7.07 | 54.04±9.25 |
| 2 | Shanghai JiaoTong University Affiliated Sixth People’s Hospital | 8 | 64.38±8.53 | 152.50±5.37 | 58.06±7.72 |
| 3 | Beijing Hospital, National Center of Gerontology | 32 | 62.24±5.42 | 158.83±5.90 | 56.25±5.17 |
| 4 | the First Affiliated Hospital of Chongqing Medical University | 65 | 62.86±6.07 | 151.85±5.77 | 54.54±6.78 |
| 5 | Western Theater Command General Hospital | 2 | 60.50±3.54 | 154.01±3.69 | 59.10±2.98 |
| 6 | the First Affiliated Hospital of Shanxi Medical University | 18 | 63.50±7.91 | 157.92±5.26 | 61.50±6.71 |
| 7 | Huadong Hospital Affiliated to Fudan University | 27 | 65.65±6.98 | 154.10±6.93 | 52.22±5.15 |
| 8 | Shanghai General Hospital, Shanghai Jiao Tong University | 25 | 64.30±6.20 | 155.57±4.78 | 53.00±5.98 |
| 9 | Nanjing Drum Tower Hospital, the Affiliated Hospital of Nanjing University Medical School | 71 | 66.21±8.33 | 152.76±6.23 | 55.96±8.00 |
| 10 | Beijing Friendship Hospital, Capital Medical University | 39 | 65.86±8.05 | 155.70±6.35 | 54.94±6.98 |
| 11 | Union Hospital, Tongji Medical College of Huazhong University of Science and Technology | 34 | 61.25±6.37 | 155.19±6.76 | 52.41±6.26 |
| 12 | Zhongshan Hospital, Fudan University | 7 | 61.00±7.77 | 154.00±2.68 | 51.97±5.53 |
| 13 | the Second Affiliated Hospital of Soochow University | 24 | 64.38±7.30 | 154.77±5.61 | 55.80±8.27 |
| 14 | Tianjin Hospital | 56 | 62.82±6.45 | 157.40±5.81 | 55.35±6.32 |
| 15 | Sichuan Academy of Medical Sciences & Sichuan Provincial People’s Hospital | 29 | 67.48±8.94 | 153.41±5.47 | 54.41±7.88 |
| 16 | West China Hospital, Sichuan University | 23 | 63.57±6.52 | 148.14±7.06 | 51.05±7.30 |
| 17 | Heibei General Hospital | 52 | 64.82±7.14 | 159.52±4.57 | 58.98±7.21 |
| 18 | The Third Affiliated Hospital of Guangzhou Medical University | 13 | 64.73±5.42 | 155.20±3.43 | 57.90±5.32 |
| 19 | Chongqing Three Gorges Central Hospital | 25 | 62.45±5.35 | 151.76±4.93 | 48.56±5.66 |

Supplementary Table 1. Baseline characteristics of participants recruited in each center

|  | Completed the study | Withdrew from the study | *P* Value |
| --- | --- | --- | --- |
|  | （n= 513） | （n= 74） |  |
| Age，year，mean ± SD | 64.26 ± 7.17 | 64.03 ± 7.45 | 0.799 |
| Nationality，n (%) |  |  |  |
| Han | 505 (98.44) | 73 (98.65） | 1.000 |
| Other | 8 (1.56) | 1 (1.35) |  |
| Height (cm), mean ± SD | 155.03±6.39 | 155.85±5.20 | 0.167 |
| Body weight (kg), mean ± SD | 55.36±7.29 | 56.03±8.60 | 0.333 |
| Time since menopause (year), mean ± SD | 15.39±7.83 | 14.61±7.63 | 0.421 |
| Previous history of fracture, n (%) | 174 (33.92) | 26 (35.14) | 0.836 |
| BMD, mean ± SD |  |  |  |
| Lumbar (L1-4) (g/cm^2^) | 0.742±0.100 | 0.712±0.107 | 0.021 |
| Total hip (g/cm^2^) | 0.709±0.095 | 0.690±0.089 | 0.092 |
| P1NP (ng/ml) | 56.85 (37.96–77.86) | 58.34 (38.23–80.45) | 0.124 |
| β-CTX (ng/ml) | 0.36 (0.23–0.51) | 0.37 (0.24–0.53) | 0.323 |
| Ca (mmol/L) | 2.35±0.10 | 2.37± 0.12 | 0.085 |
| P (mmol/L) | 1.16±0.19 | 1.19± 0.18 | 0.153 |
| ALP (U/L) | 80.48± 22.43 | 80.80± 21.49 | 0.907 |
| Cr (μmol/L) | 59.22± 10.71 | 61.43±9.90 | 0.094 |
| ALT (U/L) | 19.84± 10.11 | 22.19± 11.10 | 0.065 |

Supplementary Table 2. Baseline characteristics of participants who completed and withdrew from the study
